# Epidemiological Mapping of Canine Angiostrongylosis in Portugal: Findings from a Nationwide Prevalence Survey

**DOI:** 10.3390/vetsci12070647

**Published:** 2025-07-08

**Authors:** Beatriz Leal-Sousa, Joana Esteves-Guimarães, Jorge Isidoro Matos, Pedro Oliveira, Luís Lobo, Ana Cristina Silvestre-Ferreira, Carla S. Soares, Elena Carretón, Rodrigo Morchón, Ana Patrícia Fontes-Sousa, José Alberto Montoya-Alonso

**Affiliations:** 1 Clínica dos Gatos, 4100-207 Porto, Portugal; b.beatrizlsousa@gmail.com; 2 Clínica Veterinária Aanifeira, 4520-409 Santa Maria da Feira, Portugal; joana.eg@gmail.com; 3 Internal Medicine, Faculty of Veterinary Medicine, Research Institute of Biomedical and Health Sciences (IUIBS), University of Las Palmas de Gran Canaria, 35413 Las Palmas de Gran Canaria, Spain; jorge.matos@ulpgc.es (J.I.M.); elena.carreton@ulpgc.es (E.C.); rmorgar@usal.es (R.M.); alberto.montoya@ulpgc.es (J.A.M.-A.); 4 EPIUnit, School of Medicine and Biomedical Sciences (ICBAS), University of Porto, 4050-313 Porto, Portugal; pnoliveira@icbas.up.pt; 5 Veterinary Hospital of Porto, 4250-475 Porto, Portugal; luis.lobo@onevetgroup.pt; 6 Faculty of Veterinary Medicine, Lusófona University, 1749-024 Lisboa, Portugal; 7 Center for Animal Science Studies (CECA), Instituto de Ciências, Tecnologias e Agroambiente (ICETA), University of Porto, 4051-401 Porto, Portugal; 8 Center for Animal and Veterinary Science (CECAV), Associate Laboratory for Animal and Veterinary Science (AL4AnimalS), Department of Veterinary Sciences, University of Trás-os-Montes and Alto Douro (UTAD), 5000-801 Vila Real, Portugal; aferreir@utad.pt (A.C.S.-F.); carlasoares.medvet@gmail.com (C.S.S.); 9 VetLamaçães, Veterinary Clinic, 4715-303 Braga, Portugal; 10 CIVG—Vasco da Gama Research Center, EUVG—Vasco da Gama University School, 3020-210 Coimbra, Portugal; 11 Zoonotic Infections and One Health Group, Biomedical Research Institute of Salamanca (IBSAL), Centre for Environmental Studies and Rural Dynamization (CEADIR), Faculty of Pharmacy, University of Salamanca, 37007 Salamanca, Spain; 12 Centre for Pharmacological Research and Drug Innovation (MedInUP/RISE-Health), Department of Immuno-Physiology and Pharmacology, Veterinary Hospital of the University of Porto (UPVET), School of Medicine and Biomedical Sciences (ICBAS), University of Porto, 4050-313 Porto, Portugal

**Keywords:** *Angiostrongylus vasorum*, canine angiostrongylosis, lungworm, endemic parasitic diseases, Portugal

## Abstract

Considering the global health concern and the significant morbidity associated with canine angiostrongylosis, this study aimed to update the epidemiological profile and geographic distribution of the disease in canine populations across all continental and insular districts of Portugal, some of which were never studied before. A total of 1059 dogs were included in the study and tested for *Angiostrongylus vasorum* antigens using a commercial immunochromatographic assay. The overall prevalence was 1.13%. Higher infection rates were found in northern (3.9% in Viana do Castelo) and central (3.6% in Viseu and 3.8% in Lisbon) districts, and infection was reported, for the first time, in the districts of Leiria and Beja. The mild temperatures and elevated humidity levels, characteristic of Portugal’s northern and coastal regions, promote increased gastropod host activity and population density while also accelerating parasite development. The effect of wildlife reservoirs must also be considered, since higher seroprevalences were detected recently in red foxes from Portuguese northern regions. Bivariate Chi-square test analysis identified male sex and an outdoors lifestyle as risk factors. These findings confirm the enzootic presence of *A. vasorum* throughout the country and highlight the need for increased clinical awareness, routine screening, and the implementation of effective prophylactic strategies.

## Simple Summary

Canine angiostrongylosis is a globally distributed parasitic disease that is expanding across Europe. In Portugal, epidemiological data remain scarce, despite geo-climatic conditions that are highly conducive to parasite transmission. This study aimed to detect the presence of circulating *Angiostrongylus vasorum* antigens in dogs across mainland and insular Portugal and to identify potential risk factors for infection. Among the 1059 dogs tested, a prevalence of 1.13% was observed, with positive cases predominantly located in the north-central regions, including previously unaffected areas. An outdoor lifestyle and male sex were identified as risk factors. These findings confirm the presence of established lungworm infection and suggest a northward spread, underscoring the importance of enhanced clinical awareness and epidemiological surveillance.

## 1. Introduction

Canine angiostrongylosis (CA) is a cardiopulmonary disease caused by the metastrongyloid nematode *Angiostrongylus vasorum*. The parasite has an indirect life cycle, with domestic dogs and red foxes as the main definitive hosts and gastropods (e.g., slugs and snails) acting as intermediate hosts. Canids become infected by ingesting the third-stage larvae (L3) present in intermediate hosts, contaminated grass, or water sources. After penetration of the intestinal wall, these larvae mature and migrate through the portal circulation, reaching the right ventricle and pulmonary arteries, where they reside as adult worms. Adult females produce eggs that hatch into first-stage larvae (L1), which ascend the respiratory tract, are swallowed and excreted in the feces, infect gastropods, and continue their development into infective L3 [1].

Clinical manifestations of CA vary considerably, ranging from asymptomatic cases or mild, nonspecific signs (e.g., lethargy, weight loss) [2] to severe, life-threatening conditions such as respiratory distress, pulmonary hypertension, congestive heart failure, and hemorrhage [3,4]. The diagnosis can be challenging, not only because of these non-specific and often emergent presentations, but also because routinely used laboratory tests and thoracic radiographs commonly yield normal results [5]. A definitive diagnosis is typically made by detecting L1 in feces using direct smears or the gold-standard Baermann-Wetzel technique, and larvae may also be identified through bronchoalveolar lavage (BAL) fluid analysis [1]. In routine clinical settings, the Angio Detect™ assay enables point-of-care antigen detection from serum samples [6,7] and may overlap the time-consuming, technical, and laboring limitations of other diagnostic techniques.

Since its initial discovery in France during the 1800s, *A. vasorum* infection was thought to be confined to isolated endemic foci, such as in Denmark [8]. However, recent studies have documented a relevant geographic expansion into both newly affected and previously established tropical and subtropical regions across Europe, North and South America, and Africa [3,9,10]. In Europe, this emerging parasitic threat is characterized by increasing prevalence in wildlife reservoirs and a growing number of canine cases in areas once considered non-endemic, such as the Iberian Peninsula [11]. Over the past two decades, increased research interest and advances in diagnostic methods have contributed to a rise in published data, which reported prevalence rates in Europe ranging from 0.5% to 3% [11,12]. This broader surveillance has also underscored the previously underestimated importance of other species, such as *A. cantonensis*, known for its zoonotic potential [11]. Despite these advances, significant gaps in epidemiological knowledge persist in several countries, limiting the ability to predict the future spread of *A. vasorum* [9,11].

A study conducted in Portugal a few years ago revealed that, despite 95.5% of the surveyed dogs receiving endoparasiticides, the majority were treated with febantel-pyrantel-praziquantel combinations every 3 to 4 months, and only 6.4% received monthly prophylaxis [13]. These findings reveal a lack of awareness of certain parasitic diseases and their appropriate chemoprophylaxis protocols, which may contribute to the emergence and global spread of pulmonary nematode diseases such as CA and heartworm disease (HWD), the latter recently reported in Portugal [14].

Regarding CA, available data remain limited, with the last nationwide survey conducted nearly a decade ago in shelter dogs [15], revealing an overall antigenemia of 2.65%. Despite an overall slight decrease in prevalence between 2020 and 2022, previous reports from Spain using the same diagnostic method revealed an increase in prevalence in specific regions, such as Basque Country (from 2.74% to 3.25%) and Murcia (from 1.72% to 4.12%) [16,17]. In 2021, the first autochthonous case of CA was reported in Greece [18]. The case had a fatal outcome four days after hospitalization, with the diagnosis established only through post-mortem analysis, likely due not only to the nonspecific clinical signs presented but also to the clinician’s lack of awareness of the disease. Therefore, epidemiological surveillance and continuous monitoring of disease prevalence are crucial, not only for promoting individual pet health but also for improving the control of the spread of emerging infectious diseases.

The present study aimed to complete and update the prevalence of CA in Portugal by analyzing a large sample of domestic dogs from both continental and insular regions. Additionally, it sought to evaluate the impact of geographic, climatic, and epidemiological variables and to identify potential risk factors associated with infection.

## 2. Materials and Methods

### 2.1. Study Area and Eco-Climatic Characteristics

Portugal, the westernmost country of Southern Europe, occupies part of the Iberian Peninsula. It is bordered by Spain to the north and east and by the Atlantic Ocean to the west and south. The national territory spans 92,152 km^2^ and is divided into 20 districts, including those of the Azores and Madeira archipelagos, located in the central northern and northeastern Atlantic Ocean, respectively.

According to the Portuguese NUTS (Nomenclature of Units for Territorial Statistics) II subdivision, districts are organized into seven geographical regions: North (Viana do Castelo, Braga, Bragança, Vila Real, Porto, and parts of Aveiro, Viseu, and Guarda), Centre (Aveiro, Viseu, Guarda, Castelo Branco, Coimbra, Leiria, and parts of Santarém and Lisbon), Lisbon Metropolitan Area (Lisbon and Setúbal), Alentejo (parts of Santarém, Setúbal, and Lisbon, Évora, Beja, and Portalegre), Algarve (Faro), and the Autonomous Regions of Azores and Madeira with their respective municipalities. Coastal districts include Viana do Castelo, Braga, Porto, Aveiro, Coimbra, Leiria, Lisbon, Setúbal, and Faro, while inland districts include Vila Real, Bragança, Viseu, Guarda, Castelo Branco, Santarém, Portalegre, Beja, and Évora.

**Figure 1:**
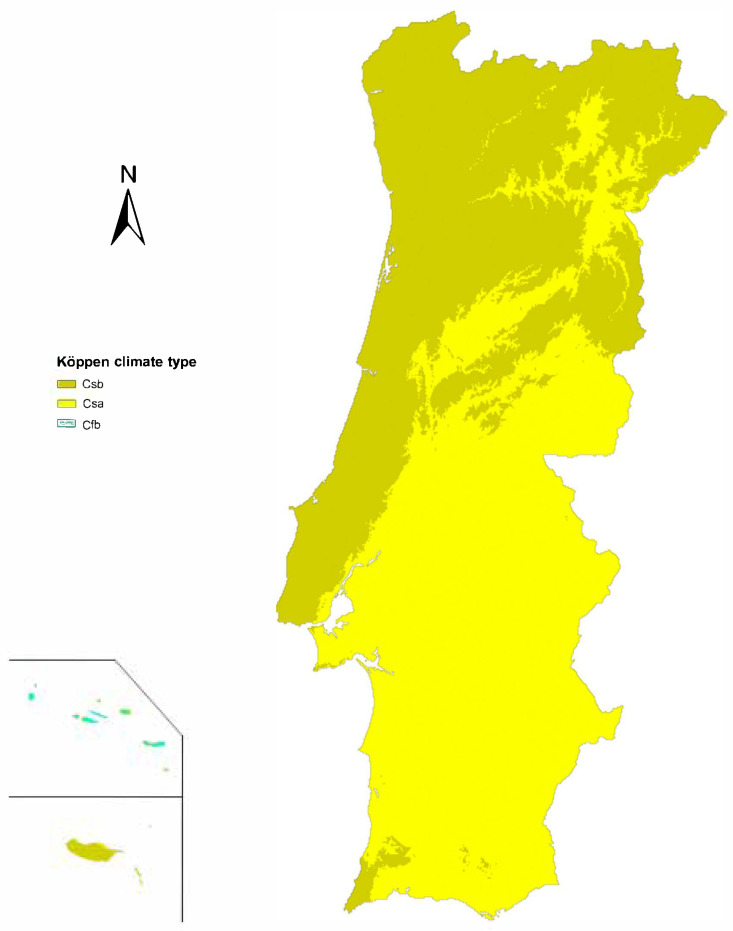
Location of different climates (Köppen Climate Classification System) in continental and insular Portugal. Legend: Csa: hot-summer Mediterranean climate; Csb: warm-summer Mediterranean climate; Cfb: temperate oceanic climate.

Mainland Portugal is predominantly characterized by a Mediterranean climate, with average temperatures during the coldest months ranging from 0 to 18 °C [19]. According to the Köppen-Geiger Climate Classification, various sub-climates are present across both mainland and insular regions, with their distribution shown in Figure 1.

### 2.2. Sample Collection and Analysis

From September 2020 to January 2025, a total of 1059 dogs were randomly selected from all Portuguese districts when presented to 36 veterinary clinics and hospitals for routine check-ups. Participation of the veterinary centers was voluntary, and written informed consent was obtained from each dog’s owner or legal guardian. The study protocol was reviewed and approved by the Ethical Committee of the University of Porto. Inclusion criteria for enrollment were as follows: (1) age over 3 months, (2) no prior history of *A. vasorum* infection, and (3) no regular prophylactic treatment against this parasite.

An a priori sample size calculation was performed using Epitools (98% confidence level, expected prevalence of 1.5%, and a precision of ±1%), resulting in a minimum required sample size of 802 dogs. As the actual sample size in the study exceeded this number, the survey provided strong statistical power to support the conclusions.

For each animal, veterinarians completed a questionnaire based on owner-reported epidemiological data. Collected information included district of origin (city and postcode), breed, diet type (homemade, mixed, or commercial), age, sex, weight, lifestyle, travel history, vaccination status, and use of prophylactic treatments. In the present study, dogs from 74 different breeds were included. Clinical signs consistent with *A. vasorum* infection (e.g., cardiorespiratory, non-specific, coagulation disorders) were recorded, along with any notable changes in biochemical and hematological parameters.

Blood samples (2–3 mL) were collected from the cephalic or jugular vein. Either fresh serum or plasma, or samples stored at 2–8 °C, were tested for the presence of *A. vasorum* antigens using a commercial immunochromatographic assay (Angio Detect^TM^ Test, IDEXX Laboratories Inc., Westbrook, ME, USA), following the manufacturer’s instructions. The test is reported to have a sensitivity of 98.1% and a specificity of 99.4%.

### 2.3. Statistical Analysis

Descriptive statistics for categorical variables were expressed as counts and frequencies. Group differences were assessed using the nonparametric Pearson’s Chi-square (χ^2^) test and Fisher’s exact test, as appropriate. For statistical purposes, dogs were categorized into four age groups: <1 year, 1–4 years, 5–10 years, and >10 years. The dog population was also stratified into seven geographic regions, according to the NUTS-II classification. Each dog’s district of origin was further classified by geographical area (inland vs. coastal, excluding islands) and climate type. Potential risk factors (i.e., sex, age, lifestyle, and climate) were assessed through bivariate χ^2^ analysis. Data were analyzed using SPSS Base 25.0 software (SPSS Inc./IBM, Chicago, IL, USA). The significance level was established at *p* < 0.05.

## 3. Results

Of the surveyed dogs, 52.9% were male and 47.1% were female. The average body weight was 20.10 Kg (±13.24). The distribution by age group was as follows: <1 year (5.6%), 1–4 years (33.6%), 5–10 years (42.9%), and >10 years (17.9%). Age data were missing for 12 dogs. Most dogs had a mixed lifestyle, living primarily indoors with regular access to outdoor environments (59.1%), followed by dogs kept exclusively outdoors (30.8%) and those living strictly indoors (10.1%). Lifestyle information was not available for three dogs. Based on demographic distribution, the sample sizes from coastal (49.5%) and inland (50.5%) areas were well balanced. The most represented regions were the Centre (33.6%) and North (26.1%), followed by Alentejo (19.6%), the Lisbon Metropolitan Area (8.7%), Algarve (6.8%), Madeira (2.8%), and the Azores (2.4%).

**Figure 2:**
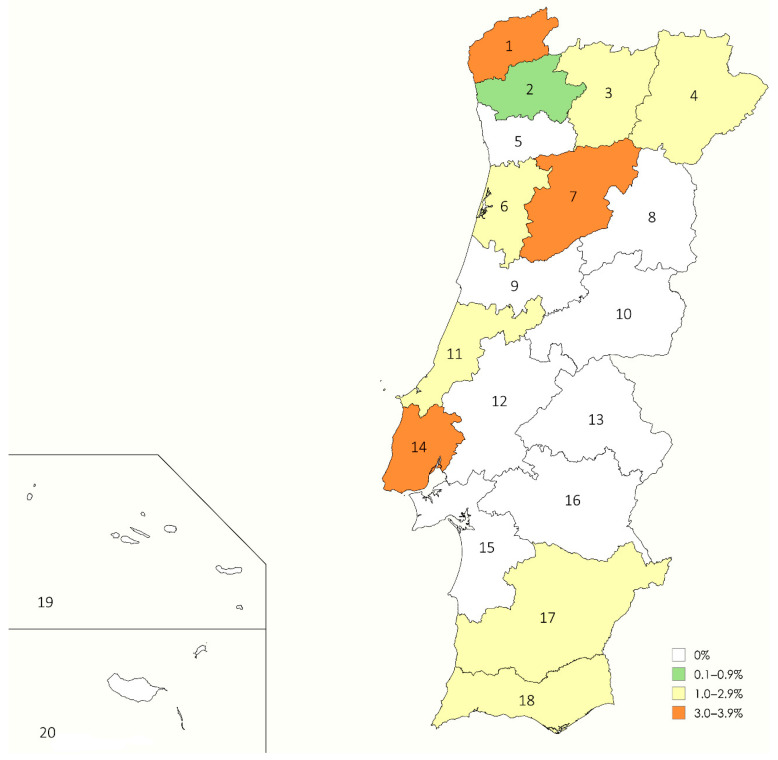
Prevalence map of *A. vasorum* infection in domestic dogs across all districts. Color gradients indicate prevalence levels, and numeric labels correspond to the districts listed in Table 1.

The overall prevalence of circulating *A. vasorum* antigens among domestic dogs in the surveyed regions of Portugal was 1.13% (12/1059), corresponding to approximately 11 positive cases per 1000 dogs. The highest prevalence rates were observed in the districts of Viana do Castelo (3.9%) and Lisbon (3.8%), followed by Viseu (3.6%), Vila Real (2.9%), Bragança and Leiria (2.0%), Aveiro (1.5%), and Beja and Faro (1.4%). No positive cases were detected in the remaining districts, including the Azores and Madeira. A detailed description of positive cases by district is provided in Table 1, while their geographical distribution and corresponding prevalence rates are illustrated in Figure 2.

The annual prevalence of *A. vasorum* was assessed over the five-year study period. In 2020, the prevalence was 1.24% (2/161), followed by 0.00% in 2021 (0/42), 0.34% in 2022 (2/585), 0.64% in 2023 (1/157), and 0.00% in 2024 (0/105). A small number of samples were analyzed in 2025 (*n* = 9), all of which tested negative. The yearly prevalence rates showed slight fluctuations, without evidence of a consistent trend over time.

In addition, the prevalence of *A. vasorum* infection was analyzed in relation to three categories—geographical area (excluding island territories), climate, and NUTS-II subdivision—as summarized in Table 2. The highest prevalence was observed in districts classified under the Csb climate type, while only four cases were reported in a Csa district. No infections were recorded in the Azores, which are characterized by a Cfb climate. Regionally, positive cases were mainly distributed across the North (*n* = 4) and Center (*n* = 4) regions. However, statistical analysis revealed no significant differences in prevalence among these categories (*p* > 0.05), likely due to limited sample sizes within subgroups.

Among the infected dogs, 7 were fed a mixed diet (commercial and homemade), 4 received a commercial diet, and 1 a homemade diet. Due to the small number of positive cases, Fisher’s exact test was applied and did not reveal a statistically significant association between diet and infection status (*p* = 0.063). Although Pearson’s chi-squared test yielded a statistically significant result (χ^2^ = 9.57, *df* = 2, *p* = 0.008), the test assumes sufficiently large expected counts, a condition not met in this dataset. Therefore, the result from Fisher’s exact test is considered more reliable in this context. Based on this, no statistically significant association was found between diet type and *A. vasorum* infection.

Moreover, the study analyzed samples from a total of 74 different dog breeds, mainly mixed-breed dogs (40.17%). The most evaluated breeds were Labrador Retriever (9.22%), German Shepherd (4.05%), Yorkshire Terrier (3.39%), French Bulldog (3.10%), Pinscher (2.82%), Portuguese Podengo (2.35%), Beagle (2.16%), Cão da Serra da Estrela (2.07%), Rafeiro Alentejano (1.6%), Jack Russell Terrier (1.6%), German Pointer (1.32%), Poodle (1.31%), and Border Collie (1.13%). No statistically significant association was found between breed and *A. vasorum* infection.

A possible association between the presence of clinical signs and infection by *A. vasorum* was assessed using Fisher’s exact test due to the low number of positive cases. Of the 582 symptomatic dogs, 8 tested positive, while 4 of the 481 asymptomatic dogs were positive. Statistical analysis did not reveal a significant association between clinical signs and infection (*p* = 0.57). Among the infected dogs, the most common symptoms were multisystemic (*n* = 6; mainly weight loss, lethargy, anorexia, lymphadenopathy, fever, or exercise intolerance), gastrointestinal (*n* = 3; vomiting, diarrhea, and melena), and cardiorespiratory (*n* = 2; cough, heart murmur, and syncope).

Also, 5 of the 12 positive dogs underwent laboratory testing (hematology and biochemistry), and the results revealed abnormalities consistent with the literature, including anemia, eosinophilia, neutrophilia, elevated liver enzymes, azotemia, and hyperglobulinemia [1,5,10].

Of the total number of animals, only 16.5% (*n* = 175) had received effective chemoprophylactic treatment against this parasite at least once within the three months prior to sampling. Among the infected animals, only one had received chemoprophylactic treatment (oral moxidectin) two weeks prior to sampling.

Potential associations between *A. vasorum* positivity and the studied variables (sex, age, lifestyle, and climate) were analyzed, and the results are presented in Table 3. Only sex and lifestyle were identified as statistically significant risk factors (*p* < 0.05). Male dogs exhibited a higher prevalence (1.8%) of infection compared to females (0.4%). The highest absolute number of positive cases was recorded in dogs living primarily outdoors. Interestingly, the prevalence was seven times higher among dogs kept exclusively indoors compared to those with both indoor and outdoor access (3.7% vs. 0.5%).

Although no statistically significant differences were observed between age groups or climate types (*p* > 0.05), the highest prevalence was recorded in dogs older than five years and in districts classified as having a Csb climate.

## 4. Discussion

Although data on angiostrongylosis infection in Portugal remain limited, regional studies support the endemic presence of the parasite across much of the country [15,20,21,22,23,24]. As early as 1999–2008, the first cases of infection in dogs from Lisbon were documented through necropsy and/or coprological analysis [22,25], and subsequent investigations reported a prevalence of 1.17% in kennel dogs from Coimbra, Santarém, and Setúbal [26], and a seroprevalence of 5% in military dogs from air bases in Aveiro, Setúbal, and Lisbon [21]. About a decade ago, the only nationwide study to date reported a seroprevalence of 0.66% and an exposure rate of 1.3% [15]. All these findings highlight the potential risk for parasite establishment in the country, particularly in central and southern districts, as well as along the western coast [15,24], possibly facilitated by its presence in wild canids such as red foxes and potentially wolves [27,28,29,30,31].

The results of the present study, with a national prevalence of 1.13%, support this theory and suggest a stable overall infection rate. Moreover, the emergence of new foci in previously unaffected districts, such as Leiria and Beja, is noteworthy. Unlike previous studies, most positive cases in this survey were detected in central-northern regions, suggesting a possible northward spread. The expansion of vector-borne diseases of veterinary relevance in Portugal has recently been reported in the case of canine HWD [14] and is a growing concern due to factors such as climate change and globalization. This trend has already been observed in Spain, a country with climatic features similar to Portugal, in both CA and *Dirofilaria immitis* infection [17,32].

Due to differences in methodologies across previous studies (e.g., sample size, type of animals, type of serological test), comparing prevalence rates over time is not feasible. Nevertheless, this study confirms the establishment of CA in central-northern districts [15], as well as in Aveiro [20,21] and Lisbon [20,21,25].

Importantly, a positive case was detected in the Algarve region, previously considered free of infection [24,33], and this study presents the first documented evidence of CA in the districts of Leiria (central Portugal) and Beja (southern coast). In contrast, the absence of positive cases in the Madeira and Azores archipelagos may be due to the limited sample size in those regions. Previous studies have only reported parasite presence in dogs from Terceira Island (Azores), with no infections identified in São Miguel or in Madeira [15,34,35].

These results align with the presence and expansion of the disease observed across Europe, primarily in regions with favorable climatic conditions for parasite development [11,36], and are similar to reported prevalence rates of 1.4–1.7% in Spain [16,17]. This is consistent with our findings, as the highest prevalences were recorded in districts characterized by warm-summer Mediterranean climates (Csb), which favor the development of intermediate hosts—except for Lisbon, which, despite its hot-summer Mediterranean climate (Csa), also showed positive cases. Lower prevalence rates in inland central and southern districts were expected due to their extreme temperatures, low vegetation, and limited rainfall [1,16,36]. Although the Azores (with an oceanic Cfb climate) showed no positive cases in this study, a previous study in Spain in regions with exclusively Cfb climates reported high prevalence, which was associated with high rainfall levels and thus an abundance of intermediate hosts [17]. Therefore, given the low number of animals tested in the Azores, our results should be interpreted with caution.

While climate plays a key role in lungworm distribution by accelerating larval development in gastropods, its recent geographic spread is driven by a combination of environmental and anthropogenic factors [11,36]. The widespread presence of *A. vasorum* is partially attributed to its ecological plasticity and ability to exploit a broad range of intermediate hosts [9]. To date, more than 25 species of terrestrial and aquatic snails and slugs have been identified as competent intermediate hosts [9,37,38]. In mainland Portugal, 195 gastropod species have been described [39], including *Arion lusitanicus* (“Portuguese slug”) [39,40], *A. ater* [40,41,42], *A. rufus* [39,41], and *Deroceras laeve* [41,42]. Notably, 8 of the 12 positive cases in northern regions corresponded to the reported distribution of *A. ater* [41], suggesting that this gastropod could be a significant vector for the parasite in Portugal. However, further studies are needed to confirm the role of *A. ater* in the transmission of CA in the country.

Although this study found that male dogs were four times more likely to be infected than females, no consistent sex-related predisposition has been established in the literature [9,43]. As expected, outdoor dogs showed a higher prevalence of infection, as an outdoor lifestyle is a known risk factor due to greater exposure to intermediate or paratenic hosts [21,43,44]. The prevalence observed in indoor dogs may be due to infections acquired during visits to parks or green areas or through contaminated food, such as raw poultry [10,45]. Therefore, periodic chemoprophylaxis is important for both indoor and outdoor dogs. In this study, 8 of the 12 infected dogs were fed home-cooked diets, although it was not specified whether raw food was included. While results did not reach statistical significance due to the low number of positive cases, further research is warranted to assess the possibility of foodborne transmission in endemic areas.

The highest prevalence in this study was observed in dogs aged five years or older, consistent with previous findings [22,25], although no statistically significant differences were found between age groups. This could reflect the low proportion of young dogs in the sampled population. Infection is generally more common in young dogs (<1 year), likely due to their immature immune systems, indiscriminate feeding habits, and scavenging behavior [5,9,16,46]. However, dogs of all ages are susceptible [2,9,47].

Unlike this study, other authors have reported breed predisposition, particularly in Cavalier King Charles Spaniels, Staffordshire Bull Terriers, Beagles, and hunting dogs [5]. The differences between studies are not entirely clear, although it is most likely that breed is not the cause of this variability, but rather the aptitude of certain breeds (for example, hunting dog breeds and guard dog breeds have greater access to the outdoors and areas of vegetation).

This study revealed a low rate of adequate chemoprophylaxis among the sampled animals. None of the positive dogs were receiving appropriate chemoprophylaxis. Only one had received a dose of oral moxidectin two weeks prior to sampling, and it is possible that the dog was already infected, with the test detecting persistent circulating antigens. Treatment guidelines for CA recommend repeating moxidectin dosing 1–2 times, 15–30 days apart [48]. The absence of recent anthelmintic treatment is a recognized risk factor for infection [9,46], and widespread misconceptions among dog owners about proper deworming frequency contribute to higher infection rates [13]. Therefore, regular administration of anthelmintic treatments is a key measure for disease control in all enzootic areas [9] and should include both topical and oral formulations containing macrocyclic lactones [49]. Additionally, measures to reduce contact with gastropods—such as discouraging scavenging behavior, feeding dogs indoors, routinely cleaning outdoor bowls and toys, and daily removal of feces—are recommended [9,10,50]. These evidence-based protocols are especially important for high-risk groups, including young dogs, dogs living in or traveling from endemic areas, and hunting breeds [9,50].

The clinical signs observed in infected dogs were consistent with those reported in the literature, as CA can manifest with a variety of clinical presentations, including respiratory, cardiopulmonary, coagulation, neurological, and, less frequently, ocular syndromes [10,46,47,51]. Therefore, CA should be included in the differential diagnosis for dogs living in endemic areas, not receiving appropriate chemoprophylaxis, and presenting clinical signs consistent with the disease.

The fact that almost all infected dogs were diagnosed during routine checkups highlights the importance of ongoing surveillance. Routine screening programs should be implemented to raise awareness among veterinarians and pet owners and to provide up-to-date information on local epidemiological risks [9,10,20,47]. Misdiagnosis remains common, often due to assumptions about the parasite’s absence in certain areas, diagnostic limitations, and cross-reactivity with *D. immitis* in antigen tests [5].

Underestimation of the true prevalence cannot be ruled out, given the parasite’s endemic nature, its increasing presence in wildlife, and the country’s favorable geoclimatic conditions, including a high abundance of competent gastropods [20,36,52]. This may be attributed to the sampling strategy, as most samples were collected in northern and central regions, and the study population consisted of healthy, owned dogs receiving regular veterinary care. Higher infection rates might have been detected with the use of additional diagnostic methods, such as the Baermann technique, antibody detection by ELISA, or molecular assays [53,54]. Nevertheless, given the large-scale nature of the study, the chosen diagnostic method was appropriate for use in veterinary clinics, providing rapid results and avoiding specialized equipment and personnel. The test used detects antigens from 9 weeks post-infection and has high sensitivity (84.6%; 97.1%) and specificity (100%; 98.4%) [6,7], although early infections may go undetected due to lower sensitivity.

A key limitation of this study was the relatively small sample size and the low number of positive cases, which limited statistical power to detect significant differences for some variables, such as prevalence by district. As a result, it was not feasible to construct an epidemiological risk map for *A. vasorum* in Portugal, as was recently done for HWD [14].

## 5. Conclusions

This study confirms the continued endemicity of *A. vasorum* in Portugal and provides new insights into its geographic distribution. A national prevalence of 1.13% was observed, with new foci identified in the districts of Leiria and Beja, as well as the first documented case in the Algarve region. Although central and southern districts have historically shown higher infection rates, our findings suggest a possible northward spread of the parasite, potentially driven by climate change and increased movement of domestic animals.

Infection was more commonly detected in male and outdoor dogs, and none of the infected animals were receiving appropriate chemoprophylaxis. These results reinforce the need for regular anthelmintic treatment in both indoor and outdoor dogs, especially in endemic and high-risk areas. Additionally, our findings support the importance of public awareness and routine screening as tools for early detection and effective disease control.

The presence of competent intermediate hosts throughout mainland Portugal, along with favorable climatic conditions, underscores the potential for further spread of the parasite. While the serological test employed was appropriate for large-scale epidemiological screening, the true prevalence of infection may be underestimated. Further studies using complementary diagnostic methods and larger sample sizes are recommended to better understand the epidemiology of CA in Portugal and to inform targeted prevention strategies.

## Figures and Tables

**Table 1:** Prevalence of *A. vasorum* in domestic dogs from Portugal, by districts, climates (Köppen Climate Classification System), and geographical area. Districts are listed in numerical order according to their corresponding regions shown in Figure 2. Abbreviations: *n*, number of sampled dogs; +, number of positive dogs; %, percentage of positive dogs; Csa: hot-summer Mediterranean climate; Csb: warm-summer Mediterranean climate; Cfb: temperate oceanic climate.

Region Number	District	Climate	Geographical Area	*n*	+	%
	Overall			1059	12	1.13
1	Viana do Castelo	Csb	Coastal	51	2	3.9
2	Braga	Csb	Coastal	59	0	0.0
3	Vila Real	Csb	Inland	34	1	2.9
4	Bragança	Csb	Inland	51	1	2.0
5	Porto	Csb	Coastal	61	0	0.0
6	Aveiro	Csb	Coastal	65	1	1.5
7	Viseu	Csb	Inland	55	2	3.6
8	Guarda	Csb	Inland	79	0	0.0
9	Coimbra	Csb	Coastal	47	0	0.0
10	Castelo Branco	Csa	Inland	56	0	0.0
11	Leiria	Csb	Coastal	50	1	2.0
12	Santarém	Csa	Inland	50	0	0.0
13	Portalegre	Csa	Inland	69	0	0.0
14	Lisbon	Csa	Coastal	53	2	3.8
15	Setúbal	Csa	Coastal	39	0	0.0
16	Évora	Csa	Inland	44	0	0.0
17	Beja	Csa	Inland	69	1	1.4
18	Faro	Csa	Coastal	72	1	1.4
19	Azores	Cfb	Insular	25	0	0.0
20	Madeira	Csb	Insular	30	0	0.0

**Table 2:** Prevalence of *A. vasorum* infection according to geographical area, climate, and NUTS-II subdivision. The information regarding the geographical area pertains solely to continental Portugal, with data from the islands being excluded. Abbreviations: *n*, number of sampled dogs or positive dogs; %, percentage of sampled dogs or positive dogs; Csa: hot-summer Mediterranean climate; Csb: warm-summer Mediterranean climate; Cfb: temperate oceanic climate; NUTS: Nomenclature of Units for Territorial Statistics.

		Test Result			
		Total	Positive		*p*-Value Chi^2^
		*n*	*n*	%	
Geographical Area	Total	1004	2	1.2	0.538
	Coastal	497	7	1.4	
	Inland	507	5	1.0	
Climate	Total	1059	12	1.1	0.658
	Csa	452	4	0.9	
	Csb	582	8	1.4	
	Cfb	25	0	0.0	
NUTS-II	Total	1059	12	1.1	0.587
	North	276	4	1.4	
	Centre	356	4	1.1	
	Lisbon	92	2	2.2	
	Alentejo	208	1	0.5	
	Algarve	72	1	1.4	
	Madeira	30	0	0.0	
	Azores	25	0	0.0	

**Table 3:** Analysis of statistically significant risk factors associated with *A. vasorum* positivity, based on studied variables (sex, age, lifestyle, and climate). Abbreviations: *n*, number of sampled dogs or positive dogs; %, percentage of sampled dogs or positive dogs; Csa: hot-summer Mediterranean climate; Csb: warm-summer Mediterranean climate; Cfb: temperate oceanic climate. * *p* < 0.05.

		Test Result				
		Total		Positive		*p*-Value Chi^2^
		*n*	%	*n*	%	
Sex	Total	1059	100.0	12	100.0	0.034 *
	Female	499	47.1	2	16.7	
	Male	560	52.9	10	83.3	
Age	Total	1047	100.0	12	100.0	0.309
	<5 years	411	39.3	3	25.0	
	≥5 years	636	60.7	9	75.0	
Lifestyle	Total	1054	100.0	12	100.0	0.010 *
	Indoor	107	10.2	4	33.3	
	Mixed	624	59.2	3	25.0	
	Outdoor	323	30.6	5	41.7	
Climate	Total	1059	100.0	12	100.0	0.658
	Csa	452	42.7	4	33.3	
	Csb	582	54.9	8	66.7	
	Cfb	25	2.4	0	0.0	

## Data Availability

The data presented in this study are available on request from the corresponding author.

## Abbreviations

The following abbreviations are used in this manuscript:

CA	Canine angiostrongylosis
L1	First-stage larvae
L3	Third-stage larvae
BAL	Bronchoalveolar lavage
ELISA	Enzyme-linked immunosorbent assay
PCR	Polymerase chain reaction
NUTS	Nomenclature of Units for Territorial Statistics
Csa	Hot-summer Mediterranean climate
Csb	Warm-summer Mediterranean climate
Cfb	Temperate oceanic climate
HWD	Heartworm disease
